# Haemolytic–uraemic syndrome with bacteraemia caused by a new hybrid *Escherichia coli* pathotype

**DOI:** 10.1002/nmi2.49

**Published:** 2014-05-27

**Authors:** P Mariani-Kurkdjian, C Lemaître, P Bidet, D Perez, L Boggini, T Kwon, S Bonacorsi

**Affiliations:** 1 Centre National de Référence associé Escherichia coli, Hôpital Robert-Debré, AP-HPParis, France; 2 IAME, UMR 1137, INSERMParis, France; 3 IAME, UMR 1137, Université Paris Diderot, Sorbonne Paris CitéParis, France; 4 Service de Réanimation, Centre hospitalier de Lons-le-SaunierLons-le-Saunier, France; 5 Service de Microbiologie, Centre hospitalier de Lons-le-SaunierLons-le-Saunier, France; 6 Service de Néphrologie, Hôpital Robert-Debré, AP-HPParis, France

**Keywords:** bacteraemia, ColV plasmid, extraintestinal virulence factors, haemolytic–uraemic syndrome, Shiga toxin-producing *Escherichia coli*

## Abstract

We describe a new atypical Shiga-toxin-producing *Escherichia coli* (STEC) responsible for a severe episode of haemolytic–uraemic syndrome in an adult with a relapse associated with bacteraemia. This STECs train of serotype O80:H2 harboured *stx2c* and *stx2d* gene subtypes, the rare *eae* ξ variant and a ColV plasmid with a conserved virulence plasmidic region involved in virulence of human and avian extraintestinal pathogenic *E. coli*. This atypical hybrid pathotype, which represents a new threat, is a further demonstration that STEC may be a recipient for extraintestinal virulence factors and raises again the question of antibiotic therapy during STEC infection.

## Introduction

Among the intestinal pathogenic *Escherichia coli*, Shiga-toxin-producing *E. coli* (STEC) are major food-borne emerging pathogens that cause bloody diarrhoea, which may be complicated by the potentially fatal haemolytic–uraemic syndrome (HUS), an important cause of acute renal failure 1. The main virulence factor of STEC is the phage-encoded cytotoxin called Shiga-toxin that exists as two main types—Stx1 and Stx2 2. In most cases, STEC also carry an enterocyte effacement pathogenicity island that causes the attaching and effacing lesions on infected epithelial cells. While other intestinal virulence factors have been described in STEC, extraintestinal manifestations are rare and virulence factors characteristic of extraintestinal pathogenic *E. coli* (ExPEC) have been rarely reported.

## Case Report

In April 2013, a 39-year-old male was admitted to the intensive care unit because of afebrile generalized tonic–clonic seizure followed by coma without focal abnormalities. White-cell count was 10 200/mL, haemoglobin was 10 g/dL and platelet count was 25 200/mm^3^ with indirect evidence of haemolysis. The electrolyte balance was normal. Cultures of cerebrospinal fluid, blood and urine were initially sterile. A computed tomographic brain scan revealed bilateral ischaemic lesions in posterior cerebral artery territory and diffuse cerebral oedema. An electroencephalographic study showed attenuation of background activity without spike-wave discharges. Furthermore, the patient received intravenous amoxicillin-clavulanate during the first 5 days of hospitalization for suspected aspiration pneumonia.

Oligoanuric acute renal failure with hypertension occurred 3 days after admission. The serum creatinine concentration increased up to 365 μmol/L. Blood tests revealed persistent thrombopenia and haemolytic anaemia (haemoglobin 6.3 g/dL) with schistocytes (3.5%). At the same time, the patient presented one episode of non-bloody diarrhoea. Stool cultures yielded a Shiga-toxin-2-producing *E. coli*, which confirmed the diagnosis of HUS. The patient required continuous veino-veinous haemofiltration (21 days) and received several erythrocyte, platelet and plasma transfusions.

Three weeks after his admission, while blood parameters were returning to normal, the patient again presented severe anaemia (haemoglobin, 6.1 g/dL) with thrombopenia (48 000/mm^3^) and schistocytes (1.7%). He remained afebrile but two blood cultures yielded a Shiga-toxin 2-producing *E. coli*. A urine culture obtained 2 days before was negative for this pathogen, indicating that urine was unlikely to be the source of bacteraemia. Intravenous antibiotic therapy with piperacillin-tazobactam and amikacin was initiated and the patient received one plasmapheresis. As the STEC strain was still detected in stools, he was treated with oral azithromycin to suppress carriage. Stools became negative 6 days after this treatment.

Ten weeks after admission, the patient was alert and oriented and ischaemic lesions had completely regressed on the computed tomographic brain scan. Serum creatinine level was 44 μmol/L, haemoglobin level was 7.3 g/dL and platelet count was 178 000/mm^3^.

## Laboratory Results

Three isolates of STEC, successively recovered from stools and blood cultures, were found to harbour *stx2c*, *stx2d*, *hlyA* and *eae* genes. The *eae* ξ variant was identified by a specific PCR 3. Enteroaggregative *E. coli* virulence factors *aggr*, *pic* and *astA* were negative. All the isolates harboured the O80 antigen (*E. coli* antisera; Statens Serum Institut, Copenhagen, Denmark) and were resistant to aminopenicillins, cotrimoxazole, nalidixic acid and kanamycin.

As the STEC strain was also isolated from blood cultures, major virulence factors of ExPEC were sought by PCR. A first screening indicated the presence of genes encoding salmochelin (*iroN*) and aerobactin (*iucC*), whose combination suggested the presence of a conserved virulence plasmidic region characteristic of ColV plasmids described in ExPEC strains 4,5. The presence of *ompT*_*p*_, *etsC*, *iss*, *hlyF*, *sitA* and *cvaA* together with *iroN* and *iucC*, which are considered to be a signature of this region, were identified in the three isolates.

**Figure 1:**
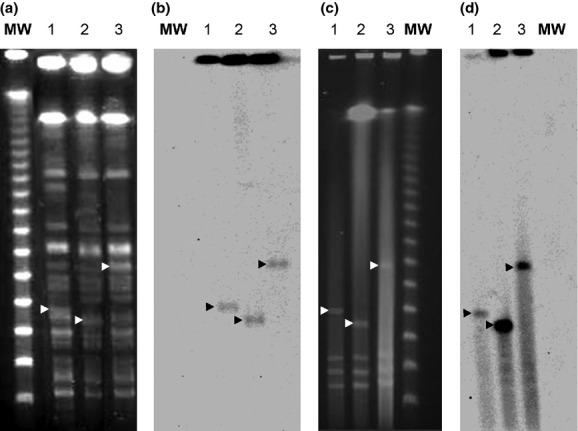
Pulsed-field gel electrophoresis of *Xba*I-restricted DNA (a) and of S1 nuclease-digested DNA (c) with the corresponding hybridizations by Southern blot (b and d, respectively) of the three Shiga-toxin-producing O80:H2 *Escherichia coli* isolates. MW, molecular weight: lane 1, first strain isolated from stool; lane 2, strain isolated from blood culture; and lane 3, second strain isolated from stool. White arrows indicate plasmids and black arrows indicate plasmid bands hybridizing with *etsC* probe.

All isolates were assigned to the phylogenetic group A with a PCR-based method and to the sequence type 301 constitutive of STc 165 using the multilocus sequence typing Achtman scheme (mlst.ucc.i.e/mlst/dbs/Ecoli). Pulsed-field gel electrophoresis of *Xba*I-restricted DNA showed that the three isolates were genetically related (Fig.1). Furthermore, oligonucleotide microarray results with 392 probes (Clondiag, Alere, France) were identical for the three strains (data not shown). Pulsed-field gel electrophoresis of S1 nuclease-digested DNA showed the presence of four plasmids for each strain; three of similar sizes in all isolates plus one of high molecular weight (ranging from 150 kbp to 300 kbp), which hybridized with *iroN* and *etsC* probes on Southern blot (Fig.1).

The main characteristics of the O80:H2 STEC strain are presented in Table1.

**Table 1:** Genetic background of the new hybrid pathotype *Escherichia coli* O80:H2

Gene	Description	Presence
*Virulence genes of Shiga-toxin-producing E. coli*		
*stx1/vtx1*	Shiga-toxin 1	Negative
*stx2c/vtx2c*	Shiga-toxin 2 variant	Positive
*stx2d/vtx2d*	Shiga-toxin 2 variant	Positive
*eae*	Intimin (adhesin)	Positive
*E-hlyA*	Enterohaemolysin A	Positive
*saa*	Shiga-toxin-producing *E. coli* autoagglutination adhesin	Negative
*efa1*	Enterohaemorragic *E. coli* factor for adherence	Positive
*tir*	Translocated intimin receptor protein	Positive
*espP*	Extracellular serine protease	Positive
*Virulence genes of enteropathogenic E. coli*		
*bfpA*	Major subunit of bundle-forming pili	Negative
*Virulence genes of enteroaggregative E. coli*		
*aggA*	Subunit of aggregative adherence fimbria AAF/1	Negative
*aggR*	Master regulator for a package of enteroaggregative *E. coli* plasmid (pAA) virulence genes, including AAF/I adherence factor	Negative
*pic*	Pic protein with mucinase activity involved in the intestinal colonization	Negative
*sepA*	SepA. *Shigella* extracellular protein	Negative
*sigA*	SigA protein, IgA protease-like homologue	Negative
*astA*	Enteroaggregative *E. coli* heat-stable enterotoxin	Negative
*Virulence genes of extraintestinal pathogenic E. coli*		
*fuyA*	Yersiniabactin siderophore (receptor)	Negative
*iroN*	Salmochelin siderophore (receptor)	Positive
*iutA*	Aerobactin siderophore (receptor)	Positive
*sitA*	Iron uptake system SitABCD	Positive
*cvaA*	Colicin V	Positive
*iss*	Increased serum survival gene	Positive
*etsC*	Type I secretion system	Positive
*hlyF*	*Α*-haemolysin (pore-forming toxin)	Positive
*ompT*	Outer membrane protein (omptin)	Positive
*mchF*	ABC transporter MchF (bacteriocin)	Positive
*sfa/foc*	S fimbriae	Negative
*papC, papGIII, papGII*	P fimbriae	Negative
*cnf1*	Cytotoxic necrotizing factor 1	Negative
*ibeA*	Endothelial invasin	Negative
*hra*	Haemagglutinin	Negative
*eitB*	ABC iron transporter	Negative
*tsh*	Temperature-sensitive haemagglutinin	Negative
*sat*	Serine protease autotransporter toxin	Negative
*vat*	Vacuolating autotransporter toxin	Negative
*cdt*	Cytolethal distending toxin	Negative
*clbN/clbB*	Colibactin	Negative

## Discussion

Here, we report the isolation of a new hybrid pathotype of STEC serotype O80:H2. A first unusual feature of this case report is bacteraemia because, to our knowledge, only six cases have been previously described during HUS 6–11. Besides rare cases of STEC infection with bacteraemia, several cases of urinary tract infection have been reported since the first report in 1996 of O103:H2 STEC 12. Among all these reports, very few searches for ExPEC virulence factors were performed.

The O157:H7 STEC, the most frequent clonal group involved in HUS does not harbour extraintestinal virulence traits. However, combined virulence pathotypes have been previously described in other serotypes. In 2011, the O104:H4 STEC, causing an outbreak of HUS in Europe, combined Shiga-toxin Stx2a production, enteroaggregative genes and two loci encoding the ExPEC siderophores aerobactin and yersiniabactin 13. The high pathogenicity island encoding yersiniabactin is known to be disseminated among intestinal pathogenic *E. coli* including certain clonal subgroups of STEC 14,15. Aerobactin has been found in more than 75% of enteroaggregative *E. coli* strains but also in several STEC 14. In contrast, the *iro* locus encoding salmochelins is extremely rare in intestinal *E. coli* 14.

This is the first time that a conserved virulence plasmidic region characteristic of ExPEC has been described in one STEC strain. This region is a key genetic determinant in ExPEC strains virulent in humans and poultry 4,5. Plasmids containing conserved virulence plasmidic regions have been especially described in neonatal meningitis strains and are associated with high levels of bacteraemia which favour the blood–brain barrier passage 5,16. The siderophores encoded by conserved virulence plasmidic region play a key role in this pathophysiological step; however, several genes of as yet unknown function are still under investigation 17,18. Our results blur the classical distinction between intestinal and extraintestinal pathogenic *E. coli*. Surprisingly, although very close in terms of virulence gene content and chromosomal backbone, the three isolates differed in their ColV plasmid size. The reasons for such apparent plasticity of the extraintestinal virulence-associated plasmid between faecal and blood isolates remain to be determined.

The serotype of our strain appears also to be atypical, as O80:H2 STEC strains have rarely been described 2,3. The European Food Safety Authority reported in their Scientific Opinion published in 2013 that O80 is an HUS-associated serotype. However, among 777 HUS cases in Europe between 2007 and 2010, only two were related to O80 STEC strains 19. The subtype of *stx2* genes in HUS-associated O80 strains has not been previously described. Stx2d has been reported to be one of the more potent toxins, similar to Stx2a 20, and may in part explain the severe presentation of our case. The potential role of the Stx2d and Stx2c combination in severe presentations remains to be studied. Interestingly, our strain harboured an infrequent intimin type that was described for the first time in two O80 STEC strains isolated from cattle 3. Moreover, examining the EcMLST database, only two *E. coli* strains of serotypes O80 and O4, respectively, belong to the sequence type 301.

Finally, our case raises again the unsolved question of antibiotic therapy during STEC infections. Here, we demonstrated that a plasmid characteristic of ExPEC could be transferred into an intestinal pathogenic *E. coli* genetic background. This plasmid probably contributes to the virulence, especially to establish bacteraemia. It is no longer recommended to treat STEC infections with antibiotics because such treatment has been associated with a higher rate of subsequent HUS 2,21. However, this novel hybrid pathotype may represent a new threat and the risk of bacteraemia may lead to a dramatic outcome in the context of HUS. Among antibiotics, it has been shown that azithromycin, in contrast to other antibiotics, is able to significantly reduce Shiga-toxin production *in vitro* 22 and to lower the frequency of long-term carriage 23. In our case, earlier azithromycin treatment may have prevented the HUS relapse.

The genetic plasticity of *E. coli* allows multiple gene combinations that result in phenotypic diversification and the emergence of new hypervirulent pathogens such as *E. coli* O80:H2. This strain may represent a threat in terms of public health requiring a redefinition of the place of antibiotic treatment during STEC infection.
